# Endothelial Cell Aging: How miRNAs Contribute?

**DOI:** 10.3390/jcm7070170

**Published:** 2018-07-10

**Authors:** Munekazu Yamakuchi, Teruto Hashiguchi

**Affiliations:** Department of Laboratory and Vascular Medicine, Cardiovascular and Respiratory Disorders, Kagoshima University Graduate School of Medical and Dental Sciences, 8-35-1, Sakuragaoka, Kagoshima 890-8520, Japan; terutoha@m3.kufm.kagoshima-u.ac.jp

**Keywords:** microRNA, Endothelial cells, Senescence, SIRT1

## Abstract

Endothelial cells (ECs) form monolayers and line the interior surfaces of blood vessels in the entire body. In most mammalian systems, the capacity of endothelial cells to divide is limited and endothelial cells are prone to be senescent. Aging of ECs and resultant endothelial dysfunction lead to a variety of vascular diseases such as atherosclerosis, diabetes mellites, hypertension, and ischemic injury. However, the mechanism by which ECs get old and become senescent and the impact of endothelial senescence on the vascular function are not fully understood. Recent research has unveiled the crucial roles of miRNAs, which are small non-coding RNAs, in regulating endothelial cellular functions, including nitric oxide production, vascular inflammation, and anti-thromboformation. In this review, how senescent-related miRNAs are involved in controlling the functions of ECs will be discussed.

## 1. Introduction

Aging is not a disease, but a series of physiological events that are usually inevitable [1,2]. In turn, aging becomes a risk factor for many diseases, such as stroke and heart failure, and accelerates age-related diseases [3]. Senescence is the biological aging of cells and represents the gradual deterioration of cellular function. Senescence of endothelial cells (ECs) impaired vascular functions, leading to aging of tissues and organ [4]. Several stimuli, including reactive oxygen species (ROS) [5,6], high glucose concentration [7], inflammatory cytokines [8], ionizing radiation [9], and telomere dysfunction [10], can induce senescence of ECs. There are several interesting signaling molecules associated with senescence in ECs [11]. For example, molecules contributing energy sensing pathways, mammalian target of rapamycin (mTOR) and AMP-activated protein kinase (AMPK), are related to the process of senescence in ECs. Endothelial mitochondrial oxidative stress is implicated in senescent vascular events and AMPK plays a defensive role in this oxidative stress in aging ECs [12]. However, the exact mechanism by which these stimuli affect the signaling pathway for senescence of ECs has not been fully understood.

One of the major small non-coding RNAs, microRNAs (miRNAs) have been studied with focus on their function as well as their unique expression in tissues and organs for the last two decades [13,14]. Many miRNAs have been evaluated as biomarkers and therapeutic targets for cardiovascular diseases [15,16]. The expression of miR-21 was upregulated in aged hearts and the miR-21-induced fibroblastic phenotype in cardiac fibroblasts [17]. miR-22 enhanced senescence of cardiac fibroblasts, which accelerated cardiac hypertrophy and fibrosis [18]. Senescence of ECs is one of the major risk factors for atherosclerosis and cardiovascular diseases [19]. Recently, several miRNAs, such as miR-217 [20], miR-34a [21], and miR-146 [22], have been identified to control the senescent process of ECs. Therefore, the role of miRNAs in ECs could be more important to regulate the onset and the process of cardiovascular diseases. We review the concept of vascular aging and senescence of ECs and endothelial miRNAs. Furthermore, the important miRNAs influencing endothelial senescence are lined up and their roles in controlling vascular aging are discussed.

## 2. Endothelial Cells: The Importance of this Silent Guardian of the Body

The structure and function of endothelial cells (ECs) are quite unique [23]. Every blood and lymphatic capillaries are covered by monolayer ECs, which possess properties of barriers to protect from invasions from the surrounding environment. ECs are spread through the whole body and the endothelium, monolayer ECs, in human can cover more than one thousand square meter of surface area throughout the body [24]. Endothelium separates systemic blood flow from underlying smooth muscle cells, fibroblasts, and pericytes and selectively connects with them by controlling the movement of fluids in blood and activated leukocytes extravasation at the place of inflammation. The function of ECs in each tissue and organ are not consistent. In arteries, the fast blood flow causes shear stress facilitates pro-atherosclerotic phenotypic change of arterial ECs [25]. In contrast, venous blood flow is relatively slow, therefore, ECs in veins are prone to augment procoagulant status [26].

Three major functions were basically classified, metabolic homeostasis, vascular hemodynamic control, and coagulation/trafficking regulation [27]. Vascular systems always need to be maintained by the balance between stimulators and inhibitors of a variety of signaling pathways. For example, pro-angiogenetic factors, such as the vascular endothelial growth factor (VEGF) inducesproliferation, migration, and activation of ECs, and anti-angiogenetic molecules, thrombospondins (TSP1 and TSP2), neutralize these effects when the level of VEGF signaling is overloaded [28]. Most ECs remain quiescent in blood flow and can proliferate in special situations. In vascular development, also called vasculogenesis, angioblast precursors differentiate into ECs and form a vascular network. Angiogenesis occurs from existing blood vessels when they get injured by inflammatory stimuli or are stimulated by tumor progression [29]. Lack of oxygen and nutrients for tumor proliferation stimulates proangiogenic signals, which mostly results in abnormal vasculatures.

## 3. Endothelial Senescence

Many stimuli, such as oxidative stress and inflammation induce and promote cellular senescence in ECs, which contribute to vascular dysfunctions. Endothelial senescence has been simply characterized by irreversible growth arrest and pro-inflammatory phenotypic change [11]. Classically, senescence has been categorized into two ways; replicative senescence [30]. Normal somatic cells cannot maintain the replicative capacity forever and go into growth arrest [31]. This has been termed by the Hayflick limit and this morphological change by the numerous cellular divisions us called replicative senescence [32,33]. In turn, exposure of oxidative stresses, hydrogen peroxide or hypoxia, induces cellular senescence, which is called stress-induced senescence [34,35]. There are some differences in messages between these two senescence states, even though they have such similar morphological alterations. In case of normal human diploid fibroblasts stimulated with hydrogen peroxide, many gene expressions have been upregulated or downregulated transcriptional factors, including heat shock protein 70 (HSP70), HSP27, cofflin 1 (COF1), and protein disulfide isomerase family A member 3 (PDIA3) [36]. 

ECs remain quiescent under physiological conditions in vascular systems. Under pathophysiological conditions such as wound healing, vascular inflammation, and tumorigenesis, ECs can replicate and proliferate, although the numbers of replication are limited. Therefore, aged ECs stop growing and senescent ECs impair their function and homeostasis in blood vessels, leading to the disruption of vascular integrity [37]. For example, senescent ECs decrease the ability of nitric oxide (NO) production. NO induces the relaxation of vascular smooth muscle cells, inhibits platelet aggregation, and blocks neutrophils/monocytes adhesion to ECs [38,39]. NO depletion leads to dysfunction of vascular homeostasis and development of hypertension, thrombosis, and atherosclerosis [40,41]. The molecular mechanisms underlying EC senescence has been intensively studied. Telomeres are repetitive DNA sequences of TTAGGG located on the end of chromosomes to protect DNA from damage. One of the machinery to maintain the telomere length is to use telomerase, a telomeric repeats synthase. A forced increase of telomerase activity by the introduction of human catalytic component of telomerase (hTERT) in cultured ECs promotes cellular transformation [42]. Addition of NO to EC-rescued replicative reduction of telomerase activity [43,44]. Oncogenic proteins such as Ras and p53 are candidate molecules responsible for ECs senescence [45]. Foreman KE et al. identified altered gene expressions in senescent ECs [46]. Plasminogen activator inhibitor-1 (PAI-1) and p21 are increased; conversely, inhibitors of differentiation of DNA binding-1 (Id-1) and Cyclin A/B1 are decreased in senescent ECs [47,48].

## 4. Endothelial miRNAs

It has been speculated that more than 60% of genes that code for proteins are regulated by miRNAs in mammalian cells [49,50]. Therefore, miRNAs can control many proteins in ECs, leading to alteration of the function of ECs. The biogenesis of mature miRNAs is complex. The primary miRNAs, generated by polymerase II, are cut by two different endonucleases, Drosha and Dicer [51,52]. When Dicer is knocked down, the cells theoretically cannot produce mature miRNAs impairing the function of miRNAs. The experiments of Dicer downregulation in vitro and in vivo have been previously performed. Knockdown of Dicer in ECs suppressed expression of miRNAs that are crucial for biological functions in ECs, such as downregulation of nitric oxide synthase [53]. Moreover, mice with EC-specific deleted Dicer exhibited an impaired postnatal angiogenic response to VEGF [54]. In contrast, the silencing of Drosha inhibited capillary sprouting and tube-forming activity in vitro less than that of Dicer and Drosha knockdown and did not alter migrating activity in ECs [55]. These findings suggested that Dicer and Drosha possess an anti-angiogenic role in ECs, however, Drosha affects EC function less than Dicer. The authors demonstrated that the discrepancy of angiogenic activity in ECs between Dicer and Drosha was due to the difference of a subset of miRNAs processed by these enzymes.

Many studies have investigated the different patterns of miRNA expression by microarray analysis, quantitative RT-PCR, and deep RNA sequencing. In mice, tissue-specific miRNAs have been examined and this study revealed a huge difference of miRNA distribution between organs [56,57]. Tissues or Organs include a variety of cells and there is accumulating data for miRNA distribution in individual cell types. A series of endothelial miRNAs have been identified and described as regulators of important genes. Notably, ECs exist in almost all tissues throughout the body. Therefore, ECs in each tissue were not really of the same phenotype and differ in terms of flow, cell–cell junction, fenestration size, and glycocalyx [58]. Systematic bioinformatics analysis unveiled genes exhibiting an EC-restricted expression pattern. Most of the genes are abundantly expressed in different tissues; eighty-five messenger RNAs (mRNAs) were identified as EC-restricted genes using HUVEC (human umbilical vein EC), HAEC (human aortic EC), HCEC (human coronary EC), HPAEC (human pulmonary artery EC), and HMVEC (human microvascular ECs) [59]. Interestingly, miRNAs that are enriched in 3’ UTR of EC-restricted genes were extracted [59]. These miRNAs have potential to change ECs phenotypes by modulating these EC-restricted genes. McCall MN et al. investigated the profiling of miRNAs from HAEC, HCEC, HPAEC, HUVEC, HMVEC, and HBMVEC (human brain microvascular ECs) and identified several miRNAs, including miR-99b, miR-20b, and let-7b, differed between these ECs [60]. Significant differences between the EC types were also shown by miRNA cluster groups; miR-17 cluster, miR-424 cluster, and miR-512 cluster, suggesting that these miRNA clusters are differentially regulated in each ECs type. For example, the expression of miR-424 cluster in microvascular ECs was higher than that in macrovascular ECs. The different expression of miR-424 in a variety of ECs might reflect the diversity of ECs functions depending on the size of blood vessels.

The methodological development of miRNA detection enabled researchers to detect miRNAs easily in any body fluid and tissue [61,62]. Accumulating evidence indicates that a variety of miRNAs are released from cells and circulate in the blood stream [63]. These cell-free miRNAs usually exist in extracellular vesicles, such as microparticles and exosomes. Some circulating miRNAs are incorporated into RNA-binding proteins, high-density lipoproteins (HDLs), nucleophosmin (NPM1), and Argonaute2 proteins (Ago2) [64,65,66]. Circulating miRNAs are also important biomarkers in patients with cardiovascular diseases [63]. Recently, aging adults have been studied for circulating miRNAs. Hooten NN et al. demonstrated that three serum miRNAs, miR-151a-3p, miR-181a-5p, and miR-1248, were decreased in old human individuals using deep RNA sequencing [67]. Senescent cells are characterized by permanent cell cycle arrest and acquisition of a senescence-associated secretory phenotype (SASP) [68]. This secretary vesicles propagate senescence to the surrounding cells and contribute to the inflammations that aid in aging [69]. Therefore, circulating miRNAs in senescent ECs are recognized as parts of the SASP.

## 5. Senescent miRNAs in ECs (Figure 1)

Several miRNAs related to the senescent process in ECs were individually discussed. Among them, the expressions of miR-126, miR-17-92, miR-23-27-24, and miR-221-222 are relatively restricted to ECs. Other miRNAs are expressed ubiquitously in many types of cells.

### 5.1. SIRT1 and miRNAs—miR-217, miR-34a, and miR-21

Sir2, identified in *S. cerevisiae*, has been recognized as a key regulator of lifespan [70,71]. At least seven human orthologs of Sir2, SIRT1-SIRT7 were discovered and SIRT1 is now the most promising molecule to impact longevity [72]. SIRT1 is a nicotinamide adenine dinucleotide (NAD)-dependent deacetylase, which mainly regulates chromatin remodeling, stress responses, and DNA repair [73]. SIRT1 plays diverse roles in controlling cancer progression by deacetylating p53, forkhead box O1 (FoxO1), and peroxisome proliferating activated receptor gamma coactivator-1 alpha (PGC-1 alpha) [74]. Whether SIRT1 serves as a tumor promoter or suppressor is still controversial, however, the importance of SIRT1 on tumor biology no more remains dubious.

In ECs, SIRT1 is an important molecule to regulate their function. SIRT1 modulates NO synthesis by controlling endothelial NO synthase (eNOS) expression. Calorie restriction induced the expression of SIRT1, which was attenuated in eNOS knockout mice [75]. SIRT1 enhanced NO production by deacetylating eNOS [76]. These suggested that the endothelial SIRT1 maintains vascular homeostasis via NO production. Moreover, molecules regulated by SIRT1, such as FoxO1 and KLF2, also play crucial roles in ECs. Acetylation of FoxO1 was increased by loss of SIRT1 negatively controlled angiogenesis [77]. SIRT1 upregulated another transcriptional factor, Krupple-like factor 2 (KLF2), inducing vasculo-protective gene expressions [78].

Several miRNAs associated with EC senescence were initially identified by Menghini R et al. in 2009 [20]. Comparison between young (population doubling levels; PDLs 8) and old (PDLs 44) HUVEC provided the list of senescence-upregulating miRNAs, including miR-217. The expression of miR-217 increased by treating HUVEC with hydrogen peroxide, which caused senescence of ECs. SIRT1 was identified as a target gene of miR-217. Since SIRT1 modulates deacetylation of FoxO1 and expression of eNOS in ECs, the increase of miR-217 might regulates the dysfunction of senescent ECs through SIRT1-FoxO1-eNOS pathway.

Overexpression of miR-34a downregulates SIRT1 expression [79]. The miR-34 family consists of miR-34a, -34b, and -34c that is located in different genes. Originally, miR-34a was reported as a tumor suppressor miRNA, and many cancers expressed a low level of miR-34a [80,81]. miR-34a attracted a great attention when p53 was proven to directly increase miR-34a. More than 50% of human cancers have p53 mutation, suggesting the importance of miR-34a in tumor progression. In fact, ectopic expression of miR-34a-induced apoptosis in many cancers [82,83,84,85]. Simultaneously, the role of SIRT1 in cancer progression has been studied, however, whether SIRT1 acts as a tumor suppressor or tumor progressor has not been elucidated yet [86]. The downstream signaling pathway of SIRT1 in each cancer might be different.

In the cardiovascular system, miR-34a was involved in apoptosis and senescence of vascular cells and heart [87,88]. Direct evidence of miR-34a in heart was provided using miR-34 knockout mice. The expression of miR-34a increased in aged mice hearts. Reduction of miR-34a inhibited myocardial infarction-induced cell death of cardiomyocytes and cardiac function in aged mice [89]. Protein phosphatase 1 regulatory subunit 10 (PPP1R10) was identified as an miR-34a target gene in this report. Delivery of antisense-miR-34a, which was knockdown of miR-34a, also improved cardiac remodeling after cardiac injury via coronary ligation [87]. In this experiment, several potential target genes of miR-34a, *Bcl2*, Cyclin D1, and *SIRT1*, were identified. These in vivo studies have clearly indicated the crucial role of miR-34a in acute myocardial infarction.

The role of miR-34a in ECs has been investigated. The expression of miR-34a increased in senescent HUVECs [21]. Overexpression of miR-34a-induced senescent phenotypes and decreased SIRT1 expression in young HUVECs. Delivery of miR-34a in endothelial progenitor cells (EPCs) increased senescence of EPCs and impaired angiogenesis via SIRT1 and FoxO1 pathway [90]. Taken together, the interaction of miR-34a and SIRT1 might be quite critical in regulating senescence in cardiovascular systems [91].

Kallistatin controls a variety of biological functions through two important domains, an active site and a heparin binding site [92]. The active site inhibits tissue kallikrein activity and increases expressions of eNOS and SIRT1. Since tissue kallikrein is one of the serine proteinase inhibitors to cleave low molecular weight kininogen, loss of Kallistatin results in hypertension and renal injury [93]. The heparin-binding site suppresses the signaling of VEGF, tumor necrosis factor alpha (TNF-alpha), and transforming growth factor beta (TGF-beta). Therefore, Kallistatin maintains homeostasis and protects from a series of pathological conditions [94]. Kallistatin exerts pleiotropic impacts on endothelial senescence. Kallistatin inhibited TNF-alpha induced senescence of endothelial progenitor cells by suppressing miR-21 and miR-34a [95]. miR-21 promotes fibroblastic alteration of ECs by TGF-beta, whose process is called `endothelial-to-mesenchymal transition (EndMT) [96]. In turn, miR-34a induced cellular senescence described above. In aorta of streptozotocin (STZ)-induced diabetic mice, Kallistatin improved vascular conditions during aging by decreasing superoxide production [95]. Kallistatin decreased the expressions of miR-21 and miR-34a decreased in aorta of this diabetic mice, which restored SIRT1 and eNOS levels. Moreover, the survival of *Caenorhabditis elegans* treated by Kallistatin under heat and oxidative stress was significantly elongated. This enhancement of worm longevity might be the consequence of reduced oxidative stress and SIRT1 regulation by miRNAs.

### 5.2. miR-126

miR-126 is one of the key ‘molecules’ to control endothelial functions. miR-126 has been identified to be the only EC-specific miRNA in vertebrates [97]. miR-126 locates in an intron of EGF-like domain 7 (EGFL7) gene, which is produced and secreted by angiogenic stimuli [98]. Knockout of miR-126 in mice caused developmental defects of vasculature, leading to an increase in embryonic lethality by systemic hemorrhage [99,100,101]. In miR-126 knockout mice, two angiogenic proteins, Sprouty-related EVH1 domain-containing protein 1 (Spred1) and a regulatory subunit of PI3K (p85 beta) were upregulated [99]. The survival of miR-126 knockout mice after myocardial infarction was reduced due to the decrease of angiogenic response [100]. In the experiment using zebrafish embryo, KLF2 regulated miR-126-modulated vascular endothelial growth factor A (VEGF-A) signaling [102]. Moreover, miR-126 inhibited vascular inflammation through vascular cell adhesion molecule 1 (VCAM1) [97]. These suggested that miR-126 controls the physiological development of the vasculature, maintains homeostasis of cardiovascular system, and protects from vascular inflammation.

In general, miR-126 (known as miR-126-3p and its complement) and miR-126-3p are expressed in ECs. Recently, both miR-126-3p and miR-126-5p have emerged as potential biomarkers for atherosclerosis [103]. The level of miR-126-3p in plasma was downregulated in patients with diabetes mellitus (DM) because the level of endothelial miR-126-3p was decreased [104]. Similarly, plasma miR-126-5p was significantly downregulated in patients with severe coronary artery disease (CAD) [105]. This study has shown that aging, one of the factors associated with cardiovascular disease, was negatively associated with the decrease of plasma miR-126-5p. Another study revealed that the miR-126 (miR-126-3p) levels in circulating blood were positively associated with the age of healthy people, however, miR-126 in patients with type 2 diabetes mellites (T2DM) did not significantly change with the age [106]. In vitro study showed that the level of miR-126 in HUVEC with high glucose was lower than that in HUVEC with normal glucose. Senescence-dependent increase of miR-126 might be a senescence-associated compensatory mechanism under non-diabetic condition.

### 5.3. miR-17-92 Cluster

The miR-17-92 cluster encodes six mature miRNAs; miR-17, miR-18a, miR-19a, miR-20a, miR-19b, and miR-92a. These miRNAs are well expressed in ECs and maintain vascular integrity [107]. Overexpression of miR-17, miR-18a, miR-19a, and miR-20b could suppress EC sprouting, and miR-92a-inhibited tube formation of ECs on Matrigel [108]. In case of ECs, the previous studies have mostly demonstrated that miR-17-92 negatively regulates angiogenesis [109]. However, there are controversies about the role of miR-17-92 in pro- or anti-angiogenic functions, since vascular features have not been investigated in miR-17-92 knockout mice yet.

Overexpression of the miR-17-92 cluster inhibited thrombospondin (TSP-1) and connective tissue growth factor (CTGF), suggesting that TSP-1 and CTGF are among of the targets of miR-17-92 cluster [110]. Expression of miRNAs in the miR-17-92 cluster decreased in aging mice heart [111]. In mice models of aging-associated heart failure, CTGF and TSP-1 were increased, causing heart remodeling; miR-17-92 disrupted oncogenic *ras*-induced senescence in primary human fibroblasts (BJ and WI38 cells) and p21 was a direct target of these miRNAs [112]. In ECs, cytokines promote senescence [113]; treatment of TNF alpha for 15 days altered the phenotype of HMVECs and one of miR-17-92, miR-20b, regulated retinoblastoma-like 1 (RBL1) [114]. Knockdown of miR-20b downregulated a cellular senescence marker, p16, indicating that miR-20b is more powerful in promoting EC senescence.

### 5.4. miR-23-27-24 Cluster

This unique cluster duplicates in two different locations; chromosome 9q22.32 and chromosome 19q13.13 (miR-23b, -27b, -24-1 and miR-23a, -27a, -24-2, respectively) [115]. The regulation of miR-23 and miR-27 is different from that of miR-24, at least, by special stimuli such as bone morphogenetic protein 2 (BMP2) [116]. Knockdown of miR-23 and miR-27 decreased VEGF induced angiogenesis, while knockdown of miR-24 in ECs increased vascularization and preserved cardiac function after myocardial infarction [117]. These three miRNAs are probably stimulated differently and work independently. Albeit the difference of only one nucleotide in the seed sequence, only miR-23b inhibited angiogenesis while miR-23a did not. Moreover, miR-23a reduced and miR-23b enhanced permeability of ECs. miR-23a and miR-23b target tight junction protein 2 (ZO-2) and junctional adhesion molecule C (JAM-C), respectively [118].

Dellago H et al. demonstrated that 12 miRNAs, including miR-23, miR-27, and miR-24, were upregulated in replicative senescent HUVECs [119]. The level of miR-23a is elevated in CAD patients and miR-23a regulates telomere shortening through telomeric repeat binding factor 2 (TRF2), suggesting that miR-23a might be able to modulate senescence [120]. miR-24 was involved in the process of senescence through targeting p16 [121], however, the role of miR-27 has not been elucidated yet. The impact of individual miRNAs from the miR-23-27-24 cluster on EC senescence should be studied in the future.

### 5.5. miR-222-221 Cluster

The cluster of miR-222-221 is highly expressed in ECs and has an antiangiogenic activity [54,122]. miR-222-221 inhibited proliferation and migration of ECs and induced apoptosis [123,124]. The expression of miR-222-221 was upregulated in balloon-injured rat carotid arteries [123]. In this model, knockdown of miR-222-221 decreased neointimal formation and increased re-endothelialization. Moreover, miR-222-221 was involved in the protective process against inflammation in ECs [125]. Target genes for miR-222 and miR-221 are quite different even though the seed sequences of both miRNAs are the same. Therefore, the functions of miR-221 and miR-222 often differ. For the function of ECs, miR-221 seems to be more important. By experiments in zebrafish, miR-221 was required for vascular development and linked to the VEGF signaling pathway [126].

The involvement of miR-222-221 in senescence of ECs has not been fully studied yet. Several genes, *ETS1*, *cKIT*, and *ZEB2*, were proven to be the targets of miR-222-221 in ECs [127]. Silencing Dicer increased eNOS expression in ECs and overexpression of miR-222-221 in Dicer knockdown EC-restored eNOS levels [54]. These data suggested that miR-222-221 might contribute to EC senescence.

### 5.6. miR-200 Family

The five miRNAs of the miR-200 family exit in two clusters. One has miR-200a, miR-200b, and miR-429 and the other has miR-200c and miR-141 in humans. Based on the observation that miR-200a level increased in the corpus cavernosum (CC) of aged rats with erectile dysfunction (ED) compared to young rats with ED, miR-200a in ECs was found to play a key role in the pathogenesis of ED in aged rats [128]. The expression of miR-200a was upregulated in cavernous endothelial cells (CECs) from aged ED rats, which influenced the downregulation of eNOS and cGMP expression. This suggested that miR-200a in senescent ECs was involved in the onset of EDs in the aged rat. Another group demonstrated that miR-200c, as well as miR-200a and miR-200b, has a key role in oxidative stress-induced apoptosis and senescence in HUVECs [129]. Introduction of miR-200c inhibited proliferation and induced senescence in HUVECs. *ZEB1* was identified as one of the target genes of miR-200c. Downregulation of ZEB1 by miR-200c enhanced senescence in ECs. Moreover, the involvement of p53 in this pathway was addressed. Activation of p53 by oxidative stress induced miR-200c expression as well as miR-200a and miR-200b [130]. Taken together, the p53–miR-200 axis might regulate senescence of ECs.

### 5.7. miR-146a

Deng S et al. revealed that miR-146a was upregulated in lineage negative bone marrow cells in aged mice, which were enriched in endothelial progenitor cells (EPCs) [131]. They identified Polo-like kinase 2 (*Plk2*) as a target gene of miR-146a. Plk2 regulates the duplication of centrosomes and stress response by genotoxic damage [132,133]. Overexpression of miR-146a enhanced senescence and augmented apoptosis, suggesting that miR-146a improves the capacity of vascular repair in EPCs.

Olivieri F et al. identified miRNAs specific for the senescent phenotype in different cultured ECs, including HUVECs, HAECs, and HCAECs [134]. The number of upregulated miRNAs in these senescent ECs was more than that of downregulated miRNAs. Highly upregulated miRNAs in aged ECs were miR-146a, miR-204, miR-367, and miR-9. The expression of miR-146a increased for 16 h after an hour treatment of hydrogen peroxide [134]. Stimulation of HUVECs with lipopolysaccharide (LPS) promoted the production of miR-146a in replicative senescent HUVECs [135]. Knockdown of miR-146a by antisense of miR-146a enhanced IRAK1 protein expression, the mediator of signaling pathway of inflammation [134]. Since pro-inflammatory conditions, such as chronic heart failure, accelerated senescence of ECs [136], the increase of miR-146a in ECs might represent senescence-associated pro-inflammatory conditions in the vasculature.

Senescence of ECs is highly associated with the increase of oxidant stress [137]. NADPH oxidase (NOX) plays important roles in regulating the production of ROS in ECs. There are many homologues of gp91phox, one of the integral membrane proteins of NOX. Among them, NOX4 is predominant in ECs [138]. The level of NOX4 was reduced by overexpression of miR-146a in HUVECs, leading to EC senescence [139]. Expression of miR-146a was decreased by the replicative senescence of ECs.

### 5.8. miR-181b

According to the profiling of replicative senescent HUVECs, the miR-181 family is quite important for senescence of ECs [134]. The level of miR-181a increased in aging HUVECs. Hori D et al. demonstrated that the expression of miR-181a in mice aorta did not change with age [140]. However, knockdown of miR-181a/b in EC significantly increased the production of nitric oxide (NO), even though the response of acetylcholine in the aortic ring of miR-181a/b knockout mice were not altered compared to wild-type mice [140]. In turn, miR-181b was one of the mechanosensitive miRNAs and upregulated in human aortic valve endothelial cells (HAVECs) sheared for 24 h under low-magnitude bidirectional shear stress (oscillatory shear stress; OS) [141]. Overexpression of miR-181b inhibited the tissue inhibitor of metalloproteinase 3 (TIMP3) in HAVECs under OS conditions suggesting that miR-181b induces ECM degradation by increased MMP activity. Although the role of miR-181b in ECs has not been studied yet, both miR-181a and miR-181b might contribute to senescence in ECs and protect from aged vascular events, such as atherosclerosis and aortic calcification.

## 6. Conclusion

Aging signs are a series of multifunctional events accompanied by structural alterations and also is usually associated with cardiovascular diseases. Endothelial senescence is directly connected to physiological longevity and the onset of diseases. EC-derived miRNAs or miRNAs affecting ECs have been studied, however, there are many questions left behind. The difference of miRNA localization or function in arteries and veins has been discussed, however, they have remained obscure. Moreover, the limitations of the in vitro studies come from the complexity of the culture of primary ECs. In vitro primary culture mostly alters the characteristics of endothelial cells. The functions and characters of ECs in culture differ from the tissues or organs where ECs are located. This review demonstrates a variety of miRNAs related to EC senescence. What is the real world of endothelial miRNAs? In theory, miRNAs function as fine tuners or buffers to gene fluctuations. However, there are still possibilities of important but unknown functions of miRNAs in ECs. Further studies are needed to unveil the role of miRNAs in regulating EC senescence.

## Figures and Tables

**Figure 1:**
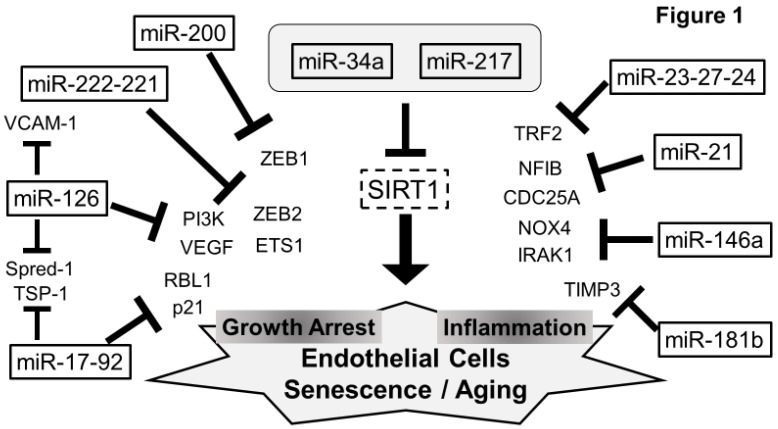
Schematic representation of endothelial miRNAs and their target in senescent ECs. The expression of miR-126, miR-17-92, miR23-27-24, and miR-221-222 are relatively abundant in ECs. In turn, miR-21, miR-181a, miR-146b, and miR-200 family, express ubiquitously in many types of cells, and influence the senescence of ECs. The increases of miR-34a and miR-217 suppress SIRT1 expression, leading to ECs aging.
